# Adenomyose utérine, étude clinique et thérapeutique: à propos de 87 cas

**DOI:** 10.11604/pamj.2015.22.73.7462

**Published:** 2015-09-29

**Authors:** Moez Kdous, Monia Ferchiou, Fethi Zhioua

**Affiliations:** 1 Service de Gynécologie Obstétrique et de Médecine de la Reproduction, Hôpital Aziza Othmana de Tunis, La Kasbah, 1008 Tunis, Tunisie

**Keywords:** Adénomyose, métrorragies, hystérectomie, adenomyosis, metrorrhagia, hysterectomy

## Abstract

Le but de notre étude est l'analyse des différents aspects épidémiologiques, cliniques, paracliniques et thérapeutiques de l'adénomyose. Il s'agit d'une étude rétrospective sur 87 patientes ayant bénéficié d'une hystérectomie pour des raisons diverses (hors prolapsus) entre le 1^er^ janvier 2000 et 31 décembre 2006 et dont l’étude histologique de la pièce opératoire a révélé la présence d'adénomyose. 586 hystérectomies (hors prolapsus) ont été réalisées pendant la même période d’étude, soit une fréquence de l'adénomyose sur pièce opératoire de 14.85%. L'age moyen de nos patientes est de 43.97 ans (extrêmes: 26-64 ans). 29.88% d'entres-elles sont ménopausées et 41.37% avaient des antécédents de traumatisme utérin. La symptomatologie a été dominée par les ménometrorragies (82.77%) et les douleurs pelviennes (71.22%). Le diagnostic préopératoire a été suspecté sur les données de l'hystéroscopie dans 63.22% des cas, de l'hystérographie dans 58.46% des cas et de l’échographie transvaginale dans 40.5% des cas. Une chirurgie radicale a été indiquée de première intention dans 57 cas, après échec du traitement médical dans 16 cas et après échec d'une réduction endométriale dans 14 cas. L'analyse histologique des pièces d'hystérectomie trouve des myomes associés dans 32.18% des cas, une hyperplasie de l'endomètre dans 13.79%, des polypes dans 5.74% et une atrophie de l'endomètre dans 3.44%. L'adénomyose, maladie énigmatique, échappe toujours à une stratégie thérapeutique bien codifiée, et demeure étroitement liée à l'hystérectomie. Son dépistage chez des femmes de plus en plus jeunes et à des stades moins avancés pourrait éviter l’évolution systématique vers les traitements radicaux.

## Introduction

L'adénomyose ou endométriose interne est définie par la présence ectopique de foyers de l'endomètre composés de glandes et de chorions profondément enchâssés dans le myomètre. Elle occupe une place importante dans la pathologie utérine. C'est une lésion bénigne mais encore mystérieuse. Depuis sa première description par Rokitansky en 1860, elle continue à soulever de nombreuses interrogations concernant sa pathogénie, mais aussi son diagnostic et ses modalités de prise en charge thérapeutiques [1]. Ce travail a pour but d’étudier le profil épidémiologique, les tableaux cliniques et les éventualités thérapeutiques de l'adénomyose.

## Méthodes

Type d’étude: étude rétrospective portant sur 87 cas d'adénomyoses utérines diagnostiquées sur pièces d'hystérectomies (hors prolapsus) et colligés sur une période de 7 ans allant du 1^er^ janvier 2000 au 31 décembre 2006, au service de gynécologie obstétrique et de médecine de la reproduction de l'hôpital Aziza Othmana de Tunis, Tunisie. L'inclusion des patientes a été faite à partir des comptes rendus d'anatomopathologie. Toutes les patientes hystérectomisées pendant la période d’étude, quelque soit la voie d'abord, et dont l'examen anatomopathologique de la pièce opératoire avait conclu à une adénomyose utérine étaient incluse dans l’étude. Le recueil des données a été fait à partir des dossiers des malades, les paramètres suivant ont été étudié: âge, gestité, parité, antécédents médico-chirurgicaux, antécédents obstétricaux, symptomatologie clinique, données de l’échographie, données de l'hystérographie et/ou de l'hystéroscopie diagnostique, traitement de première intention, indication de l'hystérectomie.

## Résultats

Nous avons réalisé durant la période d’étude 586 hystérectomies hors prolapsus, ce qui représente 14,85% d'adénomyose sur pièce opératoire. L’âge moyen de nos patientes est de 43,97 ans (extrêmes: 26 - 64 ans). Dans 72.5% des cas l'adénomyose se situe dans la tranche d’âge 40-60 ans. Les femmes ménopausées représentent 29.88% de notre population d’étude. La parité moyenne est de 4,58 (extrêmes: 0 - 15). Treize patientes sont des nullipares (14,9%), dont deux avaient une endométriose externe associée et trois avaient une adénomyose tubaire. Soixante cinq patientes (74,7%) ont accouché par voie basse et 9 patientes ont un antécédent de césarienne (10,3%). Des antécédents de traumatismes utérins ont été trouvés chez 36 patientes (41.37%), dont 26 IVG (29,88%) et 10 curetages biopsiques (11,49%). Le facteur stérilet a été retrouvé chez 25 patientes (28,37%). Les principales lésions associées à l'adénomyose, retrouvées à l'examen anatomopathologique de la pièce d'hystérectomie sont les fibromyomes dans 28 cas (32.18%), l'hyperplasie de l'endomètre dans 12 cas (13.79%), les polypes dans 5 cas (5.74%) et l'atrophie dans 3 cas (3.44%). Sur le plan clinique nous avons noté des ménometrorragies dans 72 cas (82.75%) et un syndrome douloureux dans 61 cas (71,26%). Il s'agit chez 38 patientes (43,68%) de douleurs variées à type de lombalgies, de douleurs pelviennes basses, de dysurie. Les dysménorrhées sont rencontrées dans 17 cas (19,54%), les dyspareunies dans 7 cas (8%). Nous avons noté des formes latentes dans 5 cas (5,7%). L'examen physique a retrouvé chez 28 patientes (32.18%) un utérus augmenté de volume, à contours réguliers, sensible à la palpation. Dans le reste des cas (67.82%), l'examen physique était normal. L'hystéroscopie a été pratiquée Dans tous les cas. Cet examen a confirmé le diagnostic d'adénomyose chez 55 malades (63.22%), en montrant des noyaux bleutés sous muqueux avec ou sans hypervascularisation de surface contournant les cryptes. Soixante cinq patientes ont bénéficié d'une hystérosalpingographie, elle a objectivé des images diverticulaires dans 23 cas (35.38%), une ectasie de la cavité et/ou une rigidité des cornes dans 28 cas (43.08%) et elle était normale dans 14 cas (21.54%). L’échographie pelvienne a été pratiquée chez 79 patientes soit 66,7% des cas, elle a montré un utérus augmenté de volume chez 32 femmes (40.5%) associé à un kyste ovarien dans 4 cas. La cœlioscopie lorsqu'elle a été pratiquée (20 cas), a montré des lésions d'endométriose externe pelvienne dans la moitié des cas. Le traitement a consisté en une hystérectomie avec ou sans conservation des annexes dans tous les cas. Une chirurgie radicale a été indiquée d'emblée chez 57 patientes, toutes ménopausées ou en périménopause. Les indications sont résumées dans le Tableau 1. Un traitement progestatif a été instauré de première intention chez 30 patientes avec récidive sous forme de métrorragies dans tous les cas après un délai moyen de 10 mois (extrêmes: 4 -12 mois). Dans 16/30 cas une hystérectomie de seconde intention a été pratiquée devant la persistance de la symptomatologie clinique. Dans le reste des cas (14/30 cas) une réduction endométriale a été réalisée, mais une récidive des métrorragies après un délai moyen de 18 mois (extrêmes: 9-32 mois) a nécessité le recours à une hystérectomie dans tous les cas. L'hystérectomie a été réalisée dans la majorité des cas par voie vaginale (77 cas). La voie abdominale a été réservée aux quelques cas où l'utérus était très augmenté de taille, ou lorsque un geste associé était envisageable (10 cas).

**Tableau 1:** Indications de la chirurgie radicale d'emblée

Indications	N	%
Milieu défavorisé	29	33.33%
CI au traitement médical	9	10.34
**Lésions associées**		
Utérus myomateux	12	13.79
Kyste ovarien	4	4.59
Endométriose externe	3	3.45
Total	57	65.52

## Discussion

### Epidémiologie

L'incidence de l'adénomyose est très diversement appréciée par les auteurs du fait des différentes techniques d'analyse anatomopathologiques utilisées. Sa fréquence dans la littérature varie de 5% pour Gambone [2] à 38.5% pour Bird [3] et dépend de la façon dont elle a été recherchée. L'incidence de sa découverte sur pièce d'hystérectomie varie de 5 à 70% [4]. Dans notre série la fréquence de l'adénomyose sur pièce opératoire est de 14,85%. Cette fréquence varie aussi d'un pays à un autre et d'une race à une autre, ainsi dans l’étude de Rao [5], menée chez plus de 1900 patientes, la prévalence de l'adénomyose est plus élevée chez les femmes de race noire ou d'origine indienne (18,4%) alors qu'elle est faible pour le groupe de femmes d'origine chinoise (0,9%). Si l'adénomyose touche la femme en période d'activité génitale, elle est surtout découverte en prémenopause mais aussi en postménopause. Elle paraît plus rare après soixante ans [4]. L'analyse des différentes séries publiées, retrouve la maladie dans 50 à 70% des cas dans la tranche d'age située entre 40 et 50 ans, rarement avant 30 ans et après 60 ans. Nos résultats sont conformes à ceux de la littérature, nous retrouvons la maladie dans 58,7% des cas, dans la 4^ème^ et la 5^ème^ décade (Tableau 2).

**Tableau 2:** Répartition selon les tranches d’âge (en %)

Auteurs	<30 ans	30-40 ans	40-50 ans	50-60ans	>60 ans
Molitor [6]	25	-	72.3	-	2.7
Bird [3]	-	-	55	27	18
Rochet [7]	-	6	71.4	9	-
Gautier [8]	-	15	78	7	-
Owalabi [9]	-	-	73	-	-
RAO [5]	4.7	29.7	46.1	15.4	3.8
Fukamatsu [10]	-	9.8	74.5	13.7	-
Notre série	-	22.9	58.7	13.8	4.6

L'association adénomyose et ménopause est retrouvée chez 29,88% de nos patientes. L'adénomyose est une pathologie de la multipare, le nombre moyen d'enfants par femme est variable selon les séries de 1,6 à 6,2 [10]. La parité moyenne dans notre série est de 4.58, les nullipares ne représentent que 14,90% de nos patientes, ce taux varie dans la littérature entre 6 et 20% mais il s'agit souvent de femmes jeunes avec souvent des lésions des cornes utérines et/ou une endométriose externe associée [7]. Les facteurs de risque sont représentés par les antécédents de chirurgie gynécologique. Pour certains auteurs, l'adénomyose survient préférentiellement chez des patientes dont l'utérus a subi diverses agressions [8, 11]. Dans notre série nous avons noté un antécédent de curetage biopsique dans 11,49% des cas, d'IVG dans 29,88% des cas et de port d'un stérilet dans 28,37% des cas. Le facteur césarienne ne semble pas constituer un facteur de risque pour l'adénomyose [4, 12], il n'a été retrouvé que dans 9/87 cas soit 10,34% (Tableau 3).

**Tableau 3:** Antécédents de traumatismes utérins (en %)

Auteurs	Césarienne	IVG	DIU	Curetage biopsique
Nikkanen [11]	7	-	1.47	70
GEE [13]	5.4	-	-	-
Gautier [8]	-	50	-	49.8
Harris [14]	5	-	-	-
Notre série	10.34	29.88	28,73	11.49

### Diagnostic clinique

Le diagnostic de l'adénomyose est rarement posé sur les signes cliniques. Les signes les plus couramment rencontrés dans la littérature sont: les hémorragies génitales et les douleurs pelviennes [15]. Le syndrome hémorragique: est le symptôme clinique le plus fréquent et le plus évocateur, surtout s'il est rebelle aux traitements hormonaux et au curetages hémostatiques éventuels, sans être spécifique [1]. Il est retrouvé chez 81,41% de nos patientes, ce sont généralement des ménorragies (62%) (Tableau 4). La fréquence du syndrome hémorragique est variable en fonction de l’âge, 75% des hémorragies se voient en périménopause contre 31% entre 30 et 39 ans [7]. Des formes purement hémorragiques ont été décrites dans la littérature [7]. Le syndrome douloureux: classiquement prémenstruel, il est caractérisé par une dysménorrhée: celle ci est constamment rapportée dans les différentes séries publiées avec des taux qui varient entre 20 et 40% [9, 13], elle a été notée chez 19,5% de nos patientes. Habituellement acquise et tardive, la dysménorrhée est le plus souvent primaire (76% des cas) et aggravée (40% des cas) [1]. Il semble exister une relation entre l'intensité de la dysménorrhée et le stade de pénétration dans l’épaisseur de l'endomètre. En fait, la dysménorrhée ne peut se voir que si l'adénomyose dépasse 80% de l’épaisseur de l'endomètre [16]. Néanmoins, les douleurs intermenstruelles sont fréquemment rapportées par les auteurs, dans des proportions qui varient entre 9 et 44% [7, 9]. Elles sont variées: pelviennes basses, lombaires ou urinaires pouvant égarer le diagnostic. Dans notre étude, ces douleurs ont été notées chez 43,68% de nos malades (Tableau 5). Dans certains cas, le syndrome douloureux peut être exprimé par des dyspareunies profondes. Elles ont été notées dans 8% des cas dans la série de Owalabi [9] et dans 15% des cas dans celle de Gautier [8]. Ce symptôme a été noté chez 8% de nos patientes. Aucun signe n'est spécifique d'adénomyose. Kilkku [17] à travers une enquête cas/témoins ne trouve pas de symptômes spécifiques chacun pris séparément. Par contre, le GEE [13] après avoir étudier les trois signes: douleur isolée, hémorragie isolée et douleur avec hémorragie, trouve que l'association hémorragie et douleur est significative d'adénomyose. Les formes cliniques muettes ne sont pas rares (7 à 25%). Dans notre série, la fréquence des formes latentes est de 5,7%. L'examen physique est pauvre il ne retrouve qu'un utérus augmenté de volume aux contours réguliers, souvent sensible, en particulier avant les règles [1], c'est le cas chez 32,2% de nos patientes. Les signes physiques de l'adénomyose sont peu spécifiques, comme le montre le travail prospectif de Kilkku [17] qui n'observe pas de différence entre un groupe de patientes opérées pour adénomyose et un groupe de patientes traitées pour une autre pathologie utérine bénigne. En pratique il est fréquent de noter des signes en faveur d'une pathologie associée, telle que: une endométriose externe, un fibrome utérin ou une pathologie annexielle [4].

**Tableau 4:** Syndrome hémorragique et adénomyose (en %)

Auteurs	Ménorragies	Métrorragies	Total
Molitor [6]	63	39	-
Rochet [7]	44	35	79
Owalabi [9]	-	-	63
GEE [13]	53	27	67
Bird [3]	51	13	-
Gautier [8]	-	-	75
RAO [5]	-	-	81.4
Notre série	62	20.7	81.41

**Tableau 5:** Douleur et adénomyose (en %)

Auteurs	Dysménorrhée	Dyspareunie	Autre	Total
Molitor [6]	21	-	26	-
Rochet [7]	32	-	44	75
Owalabi [9]	20	6	9	-
GEE [13]	42	-	29	-
Bird [3]	28	-	-	-
Gautier [8]	29	15	25	85
Notre série	19.54	8	43.68	71.22

### Diagnostic paraclinique

L'adénomyose est très difficile à diagnostiquer en dehors de l'examen histologique sur pièce d'hystérectomie, cependant depuis l'avènement de l'hystéroscopie, ce diagnostic est beaucoup moins flou. L'adénomyose doit être évoquée devant des kystes ou des noyaux bleutés sous muqueux avec ou sans hypervascularisation de surface localisée contenant des cryptes ou une déformation myométriale [1]. Dans notre série toutes les patientes ont bénéficié d'une hystéroscopie confirmant le diagnostic dans 63.22% des cas. L'hystérosalpingographie garde toujours sa place, elle permet d’évoquer le diagnostic dans presque 70% des cas [1]. Elle montre des images diverticulaires branchées à angle droit sur l'ombre des bords réalisant la traduction radiologique de la pénétration de l'endomètre à l'intérieur du myomètre. Leurs siège est variable, mais la localisation corporéale est plus fréquente. Ces images sont pathognomoniques de l'adénomyose mais sont malheureusement inconstantes, elles n'ont été rapportées que dans 35.38% des cas de notre série. Au niveau des trompes, l'aspect réalisé est celui d'une “boule de gui” [13]. Des signes indirects sont également notés: les ectasies diffuses à la cavité ou localisées aux cornes, la rigidité du fond utérin et/ou des cornes réalisant l'aspect de tuba erecta [1]. Dans notre série, ces aspects ont permis d’évoquer le diagnostic dans 43.08% des cas. Chez 14 de nos patientes l'hystérographie était normale soit un taux de faux négatif de l'hystérographie de 21,54%. L’échographie pelvienne, lorsqu'elle a été pratiquée dans notre série (79 cas), a permis de suspecter le diagnostic 32 fois soit 55,17%). Le diagnostic doit être évoqué devant un utérus augmenté de volume sans modification de la texture du myomètre, avec une légère diminution de l’échogénicité du mur postérieur [18, 19]. De nombreux auteurs ont montré la supériorité de l’échographie transvaginale avec une sensibilité et une spécificité de 86% [5, 20, 21]. Les critères diagnostiques retenus sont la présence au niveau du myomètre de foyers hétérogènes hypoéchogènes avec ou sans des images kystiques [22]. Le doppler trouve son intêret pour différencier adénomyose, fibrome ou sarcome, ainsi que pour guider les biopsies myométriales [23]. L'imagerie par résonance magnétique: n'a pas été réalisée chez nos patientes. Elle semble intéressante, mettant en évidence un épaississement avec irrégularité de la zone fonctionnelle et des images d'hypersignal en T2 traduisant la présence des collections sanguines et un élargissement de la bande fonctionnelle [24]. L'IRM reconnaît tous les cas d'adénomyose focale et diffuse, mais ne reconnaît pas les cas de foyers microscopiques d'adénomyose. Sa sensibilité et sa spécificité est comparable à l’échographie endo-vaginale [1]. L'imagerie par résonance magnétique est un examen coûteux, elle est réservée aux diagnostics difficiles à l’échographie. La biopsie myométriale, technique d'introduction récente n'a pas été pratiquée chez aucune de nos patientes. Elle permet la certitude diagnostique pour une adénomyose dont la pénétration ne dépasse pas les 6 mm. La biopsie se fait au niveau du mur postérieur qui est souvent le plus atteint [25]. Différents auteurs ont récemment tenté de préciser le diagnostic en réalisant des biopsies myométriales echoguidées ou par laparoscopies. La sensibilité de cet examen est située entre 40% et 70%, augmente avec le nombre des biopsies ou avec le repérage des zones suspectes, la spécificité est excellente (100%) [1]. Par ailleurs cette méthode n'est pas dénudée de risques notamment de perforation. D'autre part le geste en lui même peut être pourvoyeur d'adénomyose.

### Traitement

#### Moyens thérapeutiques

##### Le traitement médical

Il doit être utilisé en première intention d'autant plus que la femme est jeune. Les progestatifs sont largement employés en première intention. Le traitement peut être prescrit en mode séquentiel voir en continue pour bloquer l'ovulation et les menstruations pendant 3 à 6 mois. Les Norstéroides sont les progestatifs les plus employés du fait de leur action anti-oestrogénique importante. Cependant ils sont incapables de faire régresser en totalité les lésions anatomiques, ni même d'en prévenir avec certitude leur accroissement. Dans les différentes séries publiées une stabilisation ou parfois une sensible régression du volume utérin ont été rapportés mais sans disparition objective totale des lésions [4]. Le Danazol (dérivé du 17 alpha éthinyltestostérone) grâce à son activité antigonadique reste aujourd'hui le traitement le plus employé avec des résultats superposables à ceux des progestatifs. Il peut être utilisé en continu ou en séquentiel. Ses effets indésirables sont essentiellement en rapport avec son action androgénique et son effet inhibiteur de la stéroïdogénèse. Takebayashi [26] en 1995 a montré que l'utilisation locale du Danazol en intra utérin à la dose de 400 mg/j entraîne la disparition des symptômes avec régression du volume utérin et moins d'effets indisérables que la voie orale. Le Passage aux analogues LH-RH est réservé aux échecs des progestatifs ou du Danazol. Leur utilisation est cependant limitée en raison de leur coût élevé et des effets secondaires qu'ils peuvent engendrer. Malheureusement, la récidive est souvent la règle à l'arrêt du traitement médical [26]. Nous avons eu recours à un traitement médical premier chez 30 patientes (34,48%). Une récidive sous forme de métrorragie après un délai moyen de 10 mois a été notée dans tous les cas.

##### La chirurgie endoscopique

C'est une excellente alternative à l'hystérectomie totale en l'absence de lésions organiques associées. Cependant elle ne peut être proposée aux femmes désireuses de fécondité. Elle vise réaliser une endométrectomie et à obtenir une synéchie utérine complète. Trois techniques hystéroscopiques sont utilisées: la résection à l'anse: la destruction de l'endomètre est obtenue sur une profondeur de 4 mm [27]; La coagulation de l'endomètre à la Roller Ball: l'effet de destruction se situe à 3mm de profondeur [10]; la photovaporisation endométriale au laser Yag: c'est la technique la plus longue, mais qui semble être la plus adaptée à l'adénomyose, car la dévascularisation atteint 7 mm en profondeur [28]. Avec le laser Yag Goldrath [29] a traité 260 patientes, avec un résultat fonctionnel satisfaisant: 93% des patientes deviennent aménorrhéïques ou hypoménorrhéïques. La plupart des auteurs utilisent un traitement préopératoire (19-norprégnane, Danazol, agoniste LHRH) qui vise à obtenir une atrophie de l'endomètre permettant ainsi une destruction plus profonde de l'endomètre et de la partie interne du myomètre lors de l'endométrectomie. Nous avons procédé à une endométrectomie selon la technique de résection à l'anse chez 14/30 patientes après échec du traitement médical. Si les résultats sont encourageants ils sont cependant décevants, nous avons noté une récidive des métrorragies dans tous les cas après un délai moyen de 18 mois avec des extrêmes de 9 à 32 mois. La grande taille de l'utérus et la profondeur de l'atteinte myométriale peuvent constituer un facteur d’échec important [30]. Cependant, notre étude est rétrospective et pêche par ce fait d'en déduire le taux de succès de l'endométrectomie. Ce taux est rapporté dans la littérature dans une proportion qui varie entre 86% et 91% [1], mais en aucun cas le succès n'a été rapporté à la gravité de l'atteinte endométriale.

##### L'hystérectomie

L'hystérectomie totale avec conservation des annexes chez la femme jeune reste la solution de dernier recours, 96,3% de bons résultats peuvent êtres ainsi obtenu [7]. Elle est indiquée dans: les cas d'adénomyose sévère et profonde (supérieur à 1cm) ou d'utérus de grande taille; les associations pathologiques: adénomyose et fibromes utérins ou pathologie annexielle associée; les échecs des traitements médicaux et/ou endoscopiques. L'hystérectomie par voie vaginale, lorsqu'elle est possible, doit être privilégiée car elle entraîne une morbidité inférieure, des suites opératoires plus simples et une reprise de la vie professionnelle plus rapide. Elle représente la voie d'abord de choix dans notre série (88,5%). Si une annexectomie est décidée et que les ovaires ne peuvent êtres extraits par voie basse, on peut réaliser une cœlioscopie d'assistance pour pratiquer l'ablation des annexes. Dans notre série une chirurgie radicale a été indiquée d'emblée chez 57 patientes (65,52%), toutes ces patientes étaient ménopausées ou en périménopause. La majorité d'entre elles étaient issues d'un milieu défavorisé n'ayant pas pu se procurer le traitement (33.33%). Dans le reste des cas l'indication de la chirurgie radicale était une contre indication au traitement médical (10.34%) ou l'existence d'une pathologie associée telle qu'un utérus myomateux (13.79%), un kyste ovarien (4.59%) ou une endométriose externe confirmée à la cœlioscopie et résistante au traitement médical (3.45%).

### Indications du traitement

Elles dépendent de l’âge, de la pathologie associée, de la gravité des signes fonctionnels, de la taille de l'utérus et de la profondeur de la maladie [31]. Dans tous les cas les formes muettes découvertes à l'occasion d'un bilan radiologique réalisé pour d'autres motifs ne doivent pas êtres traitées [1]. -Chez les femmes jeunes âgées de moins de 40 ans ou désireuses de fécondité, le traitement sera le plus conservateur que possible. Un traitement médical s'impose en première intention et doit être poursuivi le plus longtemps possible. Le passage aux analogue LHRH est réservé aux échecs des progestatifs. La réduction endométriale, une alternative à l'hystérectomie, ne peut être considérée comme un traitement conservateur puisqu'elle compromet définitivement le pronostic de la fertilité. -Chez les femmes âgées de plus de 40 ans ou sans désir de fécondité, l'hystérectomie est la méthode de choix. C'est dans ce cadre aussi qu'il faut définir la place des techniques hystéroscopiques. En cas d'atteinte légère ou moyenne et chez les femmes désirant garder un cycle menstruel, le traitement progestatif puis la réduction d'endomètre sont d'abord utilisés [32].

## Conclusion

L'adénomyose, maladie énigmatique, échappe encore à une stratégie de prise en charge bien codifiée. Que ce soit par les difficultés diagnostiques secondaires au manque de spécificité des symptômes décrits ou par l'absence de traitement conservateur efficace. Elle demeure encore étroitement liée à l'hystérectomie, malgré le progrès réalisés dans le domaine du diagnostic par imagerie ou par biopsie myométriale autorisant l'utilisation de traitements moins radicaux. Si le progrès de nos connaissances fondamentales et ceux de la recherche thérapeutiques laissent entrevoir de nouvelles modalités potentielles pour le traitement conservateur de cette pathologie, il est raisonnable de penser que l'adénomyose doit être dépistée chez des femmes à risque, de plus en plus jeunes et à des stades moins avancés afin d’éviter l’évolution systématique vers les traitements radicaux.
